# The feasibility of vigorous resistance exercise training in adults with intellectual disabilities with cardiovascular disease risk factors

**DOI:** 10.1111/jar.12690

**Published:** 2019-12-18

**Authors:** Stijn Weterings, Alyt Oppewal, Thessa I. M. Hilgenkamp

**Affiliations:** ^1^ Department of General Practice Intellectual Disability Medicine Erasmus MC University Medical Center Rotterdam Rotterdam The Netherlands; ^2^ Abrona Healthcare Provider for people with intellectual disabilities Huis ter Heide The Netherlands; ^3^ Department of Physical Therapy University of Nevada Las Vegas NV USA

**Keywords:** adults, cardiovascular disease risk factors, exercising, feasibility, intellectual disabilities, resistance training

## Abstract

**Background:**

The cardiovascular disease (CVD) risk is high in adults with intellectual disabilities. This CVD risk can potentially be decreased with a resistance training (RT) programme at vigorous intensity, following previous research on successful High‐Intensity Training programmes. Our aim was to explore the feasibility of a vigorous RT‐programme for adults with intellectual disabilities with CVD risk factors.

**Method:**

Twenty‐four adults with intellectual disabilities with at least one CVD risk factor participated in a 24‐week RT‐programme. The training intensity was increased from novice (50%1RM) to vigorous (75%–80%1RM). Feasibility was based on the achieved training intensity at the end of the RT‐programme.

**Results:**

Nineteen participants finished the RT‐programme. Feasibility was good as 58% (11 out of 19) of the participants worked out at vigorous intensity at the end of the programme.

**Conclusions:**

It is feasible for the majority of adults with intellectual disabilities with CVD risk factors to exercise at vigorous intensity.

## 1 INTRODUCTION

The prevalence of cardiovascular risk factors, such as type 2 diabetes mellitus, hypertension, dyslipidaemia and a high waist circumference, is high in adults with intellectual disabilities (Room, Timmermans, & Roodbol, 2016; de Winter, Bastiaanse, Hilgenkamp, Evenhuis, & Echteld, 2012). These risk factors indicate an increased risk of cardiovascular disease (CVD), which is an important cause of mortality in adults with intellectual disabilities (Oppewal et al., 2018; Sobey et al., 2015). In the general population, these CVD risk factors can be positively influenced by physical activity (PA), thereby preventing the development of CVD (Colberg et al., 2010; Cornelissen & Smart, 2013; Ishiguro et al., 2016; Riebe, Ehrman, Liguori, & Magal, 2018; Umpierre et al., 2011). However, the proportion of adults with intellectual disabilities who participate in PA consistent with public health recommendations is low (Hilgenkamp, Reis, van Wijck, & Evenhuis, 2012; Peterson, Janz, & Lowe, 2008). Increasing PA through training and exercising may, therefore, be an effective strategy to reduce CVD risk and prevent the development of CVD in this population.

Traditionally, aerobic training (AT) is recommended to reduce CVD risk, but nowadays resistance training (RT) is also recommended (Colberg et al., 2010; Riebe et al., 2018). Both AT and RT can be customized to the capabilities of the participants to assure optimal participation, safety and effectiveness. However, RT has potential additional benefits compared with AT. Firstly, RT seems to be a more attractive way of exercising than AT for overweight and sedentary individuals (Holten et al., 2004). In line with that, compliance rates have been shown to be higher in RT‐programmes than in AT‐programmes in sedentary older adults (Dunstan et al., 2002; Hong, Hughes, & Prohaska, 2008). Secondly, RT has a double positive impact on the resting metabolic rate, because RT results in more muscle mass which requires more energy at rest, and RT causes micro‐trauma in muscle tissue that requires energy for the muscle remodelling processes (Westcott, 2012). Finally, an increase in muscle strength has positive effects on performing activities of daily living (ADL) and is beneficial for reducing sarcopenia (age‐related loss in muscle mass), which is already prevalent at a young age in people with intellectual disabilities (Bastiaanse, Hilgenkamp, Echteld, & Evenhuis, 2012; Beltran Valls et al., 2014; Savage et al., 2011). These advantages of RT make it interesting to explore the potential of an RT‐programme for adults with intellectual disabilities at risk for developing CVD.

From studies in the general population we know that to effectively reduce CVD risk with an RT‐programme, it is necessary to train all large muscle groups at vigorous intensity (Riebe et al., 2018). Furthermore, a gradual increase in training intensity is advised for novice trainees, until vigorous intensity is reached (Riebe et al., 2018). Besides these general recommendations regarding RT, there are some special considerations to take into account when exercising with adults with intellectual disabilities. Adults with intellectual disabilities can have motivational problems, often use medication that might impede being physically active, often have motor control issues, generally have a shorter attention span and often do not want to continue exercising when there is a physical discomfort (Bossink, van der Putten, & Vlaskamp, 2017; Riebe et al., 2018). These considerations influence the feasibility of an RT‐programme for adults with intellectual disabilities.

Studies regarding RT‐programmes specifically for adults with intellectual disabilities are scarce, and no studies have been performed in adults with intellectual disabilities with CVD risk factors. Most studies performed a combination of RT and AT or RT and balance exercises (Calders et al., 2011; Carmeli, Zinger‐Vaknin, Morad, & Merrick, 2005; Mendonca, Pereira, & Fernhall, 2011; van Schijndel‐Speet, Evenhuis, van Wijck, van Montfort, & Echteld, 2016). Some studies only focused on people with Down syndrome (DS) and not intellectual disabilities in general (Mendonca et al., 2011; Shields, Taylor, & Dodd, 2008). People with DS have syndrome‐specific mental and physical problems, such as hypotonia and ligament laxity, which impairs the generalization of these results to people with intellectual disabilities in general. Also, most studies did not report training intensity (Machek, Stopka, Tillman, Sneed, & Naugle, 2008; Podgorski, Kessler, Cacia, Peterson, & Henderson, 2004; van Schijndel‐Speet et al., 2016). The studies that included resistance exercise at moderate to vigorous intensity focused only on healthy adults with mild intellectual disabilities (Calders et al., 2011; Carmeli et al., 2005; Podgorski et al., 2004). It is therefore not known whether RT at vigorous intensity is feasible for adults with mild and moderate intellectual disabilities with CVD risk factors.

A feasibility study is most suited to address this question (Eldridge et al., 2016; Thabane et al., 2010). Therefore, the aim of this study was to explore the feasibility of a 24‐week RT‐programme progressing to a vigorous training intensity for adults with intellectual disabilities with CVD risk factors. Additionally, this study aimed to examine the dropout, attendance, safety and experience of the participants, as well as the experience of trainers of the 24‐week RT‐programme. We expected that our RT‐programme is feasible for adults with ID, because it is an individualized programme fitted to the possibilities of each participant, provided by expert trainers, with a focus on safe execution, progression and motivation.

## 2 METHODS

### 2.1 Study design

This multicentre observational feasibility study was part of the “Healthy Aging and Intellectual Disabilities” (HA‐ID) consortium; a consort of three care providers for people with intellectual disabilities in the Netherlands; Abrona (Huis ter Heide), Ipse de Bruggen (Zoetermeer) and Amarant (Tilburg) in collaboration with the Chair for Intellectual Disability Medicine of the Erasmus MC, University Medical Center Rotterdam (Hilgenkamp et al., 2011).

### 2.2 Participants

All participants lived and/or worked in a residential or community‐based setting of the participating care providers for people with intellectual disabilities in the Netherlands. Individuals with intellectual disabilities within the residential setting were invited to participate by their nurse practitioner if they were diagnosed with a mild (IQ = 50–69) or moderate (IQ = 35–49) intellectual disability, older than 18 years and diagnosed with at least one CVD risk factor (type 2 diabetes mellitus, hypertension, hypercholesterolaemia and/or overweight/obesity). Participants were excluded when physical problems inhibited exercising or when there was no medical clearance given by the physician. All participants or their legal representatives gave written informed consent. This study was performed in accordance with the Helsinki declaration (WMA, 2013). The medical ethics committee of the Erasmus MC, University Medical Center Rotterdam, the Netherlands, approved this study (MEC‐2016–574).

### 2.3 RT‐programme

#### 2.3.1 Training sessions

The participants completed a 24‐week RT‐programme, with two training sessions a week (48 sessions in total). Each session lasted approximately 60 min and started with a 5‐min warm‐up of low intensity aerobic activity (cycle ergometer or treadmill), after which the resistance exercises were performed. Each session ended with cooling‐down and stretching exercises for 5 min. The participants were supported and supervised by a physiotherapist or physical activity instructor during the entire programme to ensure good posture, safety and support. An instruction session at the start of the programme was provided for all trainers to make sure they understood the training protocol and exercises and were able to execute them correctly. The trainer‐participant ratio was 1:1 (*n* = 8), 1:2 (*n* = 12) or 2:4 (*n* = 4), depending on the participant's preferences to train individual or in a group, and depending on the organisational possibilities (training time and day, location, availability of trainers). The training sessions were performed at different locations, either at a local physiotherapy practice (*n* = 13), at home (*n* = 1), or at a local fitness centre (*n* = 9). Both the trainer‐participant ratios and training locations are feasible options in daily practice and therefore used in this study.

#### 2.3.2 Exercises

The RT‐programme consisted of seven exercises (step up, push off/up, seating squat, abdominal curl, bridge pose, biceps curl and triceps curl). In our previous pilot study, these exercises had been recommended by experienced physiotherapists and physical activity instructors working with adults with intellectual disabilities and found feasible to perform (Weterings, Oppewal, van Eeden, & Hilgenkamp, 2018). However, the RT exercises were not set in stone; when necessary trainers could tailor the exercises to the physical capabilities of the participant (Weterings et al., 2018). Researchers were available to provide the trainers with feedback throughout the programme when adapting an exercise of the RT‐ programme to make sure the participant performed a complete workout as intended.

#### 2.3.3 Progression in training intensity

Most participants were novice trainees, and therefore, the RT‐programme had five phases, with increasing training intensity in each phase (see Table 1) (Riebe et al., 2018). Each phase consisted of at least eight sessions, so the bodies of the participants could adapt to the physical strain of the exercises in order to prevent injuries. To move to the next phase, at least five out of the seven exercises should be performed with good posture and breathing technique during eight training sessions. The first phase was the familiarization phase, in which the participants were introduced to the exercises, training posture and breathing techniques.

**Table 1:** Training intensity per phase

Phase	% of 1RM	No. of sets	No. of repetitions	Rest between sets
Familiarization	50	2	20	30 s
1	60	2	18	30 s
2	70	3	12	1 min
3	75	3	10	1 min
4	80	3	8	2 min

Note

1RM: the maximum amount of weight that a person can possibly lift for one repetition over the whole range of motion.

The training intensity was described as the percentage of an one repetition maximum (1RM), which is “the greatest resistance that can be moved through the full range of motion in a controlled manner with good posture” (Riebe et al., 2018). For safety reasons, there was no 1RM‐measurement of each exercise at the start of the programme, because training posture and breathing techniques were not trained yet. Instead, the trainer selected a weight for each exercise with which he/she expected that the participants could perform a maximum of 20 repetitions (exercising at 50% 1RM), which was then set as number of repetitions during the familiarization phase. The participants were asked to work to tolerance or until the intended repetitions were reached. After this starting point, whenever participants performed two sets of the intended repetitions in a controlled manner with good posture and breathing technique, the training weight was increased by ∼5% or the smallest amount possible for each exercise, without changing the number of repetitions (Riebe et al., 2018). As participants moved on to the next phase, the number of intended repetitions decreased to correspond with the level of %1RM (see Table 1) while the training weight was increased, to make sure weight and intended repetitions corresponded with the 1RM‐score of the previous training session. The trainers logged the intended training intensity, training weights and performed repetitions of each training session, exercise and set. Within each phase, The recovery between sets was between 30 s and 2 min depending on the training intensity (see Table 1), in accordance with the ACSM guidelines for RT (Riebe et al., 2018).

### 2.4 Measurements

#### 2.4.1 Participants' characteristics

Age, sex, the presence of CVD risk factors (type 2 diabetes mellitus, hypertension, dyslipidaemia and/or overweight/obesity), and diagnosis were derived from medical records. Behavioural therapists or psychologists categorized level of intellectual disabilities as mild (IQ = 50–69) or moderate (IQ = 35–49) for each participant. Body mass index (BMI) was calculated by weight (measured with Seca Robusta type 813, in kilogram) divided by squared height (measured with Seca 216 height rod, in metre). Waist circumference was measured with a flexible tape (in centimetre). All measurements were performed at the start of the RT‐programme.

### 2.5 The feasibility of training at vigorous intensity

The achieved training intensity at the end of the RT‐programme was used to define feasibility. Vigorous intensity was defined as training intensity of at least 75%1RM. Feasibility of the RT‐programme was characterized as low (≤25% of participants reached ≥75%1RM), moderate (>25% and ≤50% of participants reached ≥75%1RM), good (>50% and ≤75% of participants reached ≥75%1RM) and excellent (>75% of participants reached ≥75%1RM) (Hilgenkamp, van Wijck, & Evenhuis, 2013).

### 2.6 Dropout

The dropout was presented as the percentage of participants not finishing the 24‐week RT‐programme. The researcher logged the dropout after consulting the participant, the trainer and the participants' caregiver. The results of the participants that dropped out were not further used for analyses of this study.

### 2.7 Attendance, safety, participants' experience and trainers' experience

The trainers logged attendance and adverse events of each training session. At the end of the programme, a custom‐made questionnaire was used to evaluate the participants' experience and trainers' experience. The participant's questionnaire contained questions for the participants about the experience, difficulty and acceptance of the RT‐programme. The participants responded mostly on a 5‐point Likert scale, but some questions were open questions, so participants could give feedback in their own words. The trainers' questionnaire contained open questions regarding the RT‐programme and their take on the difficulty and acceptance of the RT‐programme by the participants.

### 2.8 Statistical analysis

The participant's characteristics were analysed for all participants with descriptive statistics. The training intensity, dropout, attendance and participants’ experiences of all participants who finished the 24‐week RT‐programme were analysed with descriptive statistics. The trainers’ experience was analysed with descriptive statistics. The additional comments of the participants and trainers were described qualitatively. The data were analysed by using SPSS version 24 (IBM Corporation).

## 3 RESULTS

### 3.1 Participants' characteristics

Twenty‐four participants (13 women/11 men) with mild (*n* = 13) and moderate (*n* = 11) intellectual disabilities started the RT‐programme. Seven participants had type 2 diabetes mellitus, seven had hypertension, five had dyslipidaemia and 18 were diagnosed as being overweight or obese. However, our baseline BMI measurement revealed that 22 participants were overweight/obese, one participant was slightly underweight and one had an average BMI (see Table 2).

**Table 2:** Participants’ characteristics

Number of participants	24
Male	11 (45.8%)
Female	13 (54.2%)
Level of ID	
Mild	11 (45.8%)
Moderate	13 (54.2%)
Diagnoses	
Down syndrome	3 (12.5%)
Cerebral Palsy (GMFCS I)	2 (9.5%)
Age (years), mean ± *SD* [range]	44 ± 17 [23–75]
CVD Risk	
Diabetes mellitus, type 2	7 (29%)
Hypertension	7 (29%)
Dyslipidaemia	5 (20%)
Overweight/Obese	22 (92%)
BMI ± *SD* [range]	33.9 ± 6.9 [17.4–44.2]
Underweight	1 (4%)
Normal	1 (4%)
Overweight	5 (21%)
Obese	5 (21%)
Severe obese	5 (21%)
Morbidly obese	7 (29%)
Waist circumference, mean ± *SD* [range] in cm.	115 ± 15 [82–144]

Abbreviations: BMI, body mass index; cm, centimetre; CVD, cardiovascular disease; GMFCS, Gross Motor Function Classification Score; ID, intellectual disability; *SD*, standard deviation.

### 3.2 Feasibility of training at vigorous intensity

**Figure 1:**
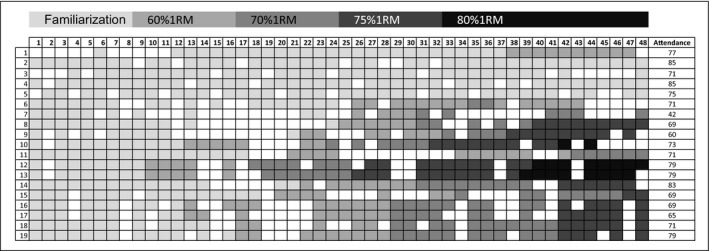
Training intensity of the participants per session. The participants who finished the RT‐programme are listed on the left. The coloured squares show the training intensity of each session, and the blank squares show the training sessions that were missed. The attendance of each participant was noted on the right in percentages

Nineteen participants finished the 24‐week RT‐programme, and 11 out of 19 participants (58%) worked out at vigorous intensity, of which eight at 75%1RM (42%) and three at 80%1RM (16%) at the end of the programme. Therefore, the feasibility to train at vigorous intensity was good for adults with intellectual disabilities. Four participants did not exceed the lowest level of training intensity (familiarization, 50%1RM), due to difficulties with increasing training weight. They had difficulties performing an exercise every time the training weight was increased. This prevented the trainer to increase the training weight and thereby the intensity. Two participants worked out at 60%1RM and two participants at 70%1RM at the end of the programme (see also Figure 1).

#### 3.2.1 Dropout

Five participants (21% of participants, 2 men) did not finish the 24‐week RT‐programme. Three participants did not want to continue training due to motivational problems, and two participants stopped due to injuries not related to the programme (one participant tore his knee ligaments and one participant fractured his hip).

### 3.3 Attendance

The overall attendance for the participants who finished the programme was 73% (an average of 35 sessions per participant, range 40%–91%, see Figure 1). Training sessions were cancelled due to holidays (36% of the cancellations), absent trainers (11%) and cancellation due to force majeure (fire alarm and cancellation due to a storm warning, 11%). Without the cancellations due to holidays, absent trainers and force majeure, the attendance would be 85%. Other reasons for cancellation were illness of the participant (15%), forgotten (4%), did not want to train (4%) and not reported (19%).

### 3.4 Safety

Other than some muscle soreness after training, no adverse events related to the RT‐programme were reported.

### 3.5 Participants' experience

Most participants (*n* = 18) liked participating in the RT‐programme and would recommend joining the RT‐programme to other people. One participant did not understand this question and did not respond. For most participants (*n* = 18), the 24‐week duration was not too long and they would join again (see Table 3). Eight participants noticed a better performance in daily life after the RT‐programme (see Table 3 for their comments).

**Table 3:** Responses of the participants about their experience with the RT‐programme

	Positive	Neutral	Negative	Remarks
Did you like to participate in the RT‐programme?	18	1		No remarks
Did you like to train at your achieved intensity?	17	2		No remarks
Would you join the RT‐programme again?	13		6	No remarks
Would you recommend joining the RT‐programme to other people?	13		5	1 participant did not understand the question
Duration of 24‐week RT‐programme	14		1 too long 4 too short	No remarks
Did you like to train 2x per week?	14	3	2	No remarks
Did you notice a difference in daily life after/during the RT‐programme?	8	11		“Walking is easier” “My diabetes is stable for the first time” “It is easier to do my daily chores” “I can lift heavy boxes at work now” “Cycling is easier now” “I feel better after training”

Abbreviation: RT, Resistance training.

### 3.6 Trainers' experience

The trainers responded that all participants were able to perform the RT‐programme. Even though six participants needed continuous feedback on the execution of the exercises and three participants found it difficult to perform one or two of the exercises correctly. These exercises were performed too fast or not in the full range of motion. Two participants found it difficult to handle increasing weights during training. For six participants, the trainers had to make adjustments to the exercises to meet their physical possibilities, for example some participants could not perform the bridge pose, which was adapted to a lateral pull down or a back raise; one participant used the leg raise instead of the abdominal curl; and one participant could not perform push ups because of arm length differences and the seated horizontal push was used. Throughout the programme, close supervision of the trainers was necessary to ensure good posture and breathing technique. One trainer had to split the training sessions from a group (1:2) to individual training (1:1), because of the negative interaction between the participants.

## 4 DISCUSSION

The results of this study showed that 58% (11 out of 19) of the participants achieved vigorous training intensity of ≥75%1RM. Therefore, the feasibility to exercise at vigorous intensity was considered good for adults with mild or moderate intellectual disabilities with CVD risk factors. Although the feasibility was good, the overall number of training sessions at a vigorous intensity was lower than expected, which could limit potential health benefits. The step‐by‐step increase in training intensity, which is advised for novice trainees (Riebe et al., 2018), took half of the training sessions before vigorous training intensity could be reached. This, combined with the fact that 27% of the training sessions were cancelled and that many participants progressed more slowly than expected through the different intensity phases, led to a limited time of training at vigorous intensity or to not reaching vigorous intensity at all in 24 weeks.

Feasibility was anticipated to be negatively influenced by motivational problems and motor control problems many adults with intellectual disabilities experience, and because adults with intellectual disabilities find it often difficult to continue exercising when there is a physical discomfort (like pushing through resistance, sweating, a raised heartbeat or breathing heavily) (Bossink et al., 2017; Riebe et al., 2018). Therefore, we tried to increase feasibility by working with trainers with a lot of experience in working with adults with intellectual disabilities. The trainers were experienced physiotherapists and physical activity instructors, and all trainers received an instruction session before the start of the programme to ensure understanding of the protocol. These trainers were able to adapt exercises, when necessary, to the possibilities and limitations of the participants and ensured that the participants maintained a correct posture during exercising. They also motivated the participants to do their best and perform at the required intensity, all while making sure there was a positive atmosphere during training through humour and positive reinforcement. Furthermore, the RT‐programme started with a familiarization period tailored to each participant.

Eighteen participants liked the RT‐programme, 13 would join again and just 4% of the training sessions were cancelled because the participant did not want to train. Therefore, motivation of the participants for the RT‐programme seemed no problem. It seemed more difficult for adults with intellectual disabilities to perform the RT‐programme, considering the result that many participants progressed more slowly through the different intensity phases than expected. The participants needed more time to get used the exercises and the exercising, even though the trainers provided close support and supervision. Furthermore, three participants found one or two exercises too difficult to perform during the whole RT‐programme, two participants found it difficult to handle increasing weights and for six participants alternative exercises had to be used. Therefore, these motor control problems and the physical discomfort during training had a direct impact on the feasibility of the RT‐programme.

To increase the feasibility, many facilitators mentioned in the literature were used in this study to help the participants to continue training in the RT‐programme (Bossink et al., 2017; Riebe et al., 2018; Weterings et al., 2018). The trainers tried to create a positive and comfortable atmosphere during training; the participants received a diploma and medal at the end of the programme; there was often social interaction with peers; there was always guidance during training from the trainer; and the RT‐training was organized close to home.

There was a dropout of 21% (5 out of 24). Two studies, one on RT in adults with a mild intellectual disabilities (Calders et al., 2011) and one study on RT in adults with DS (Shields et al., 2008), had no dropouts and another study of older adults with borderline to profound intellectual disabilities had a comparable dropout of 20% (3 out of 15) (Podgorski et al., 2004). Adults with intellectual disabilities are a heterogenic population with many motivational, behavioural and physical problems. A dropout can occur, despite all efforts to support the participants during training. In our study, five participants dropped out, three due to motivational problems and two due to injuries not related to the RT‐programme.

For this feasibility study, we also wanted to know how many of the 48 sessions would be attended, to be able to anticipate the attendance in our following effect study. The average attendance was 73%, which was lower than the study of Calder et al., (>90% out of 40 sessions in 20 weeks) (Calders et al., 2011) and of Shields et al., (92.8% out of 20 sessions in 10 weeks) (Shields et al., 2008), but comparable to the study of Podgorski et al., (75% out of 48 sessions in 12 weeks) (Podgorski et al., 2004). A review on RT in adults with type 2 diabetes mellitus in the general population showed an attendance of 75%–100% across studies (Umpierre et al., 2011) and a review on the effect of RT on CVD risk factors in overweight/obese children showed an attendance of 76%–96% across studies (Dietz, Hoffmann, Lachtermann, & Simon, 2012). The attendance of our study is comparable with the lower end of the studies mentioned in both reviews. Future studies should anticipate that around 25% of the training sessions will be cancelled and compensate with extra training sessions and/or try to increase attendance. There are some ways to increase the attendance. Future studies can reschedule a new training session when a participant cannot be present and need to make sure there will always be a trainer present. Furthermore, participants (and caregivers) should be reminded that the participant has a training session scheduled, to further increase the attendance.

The participants in this study had a large variation in age, type of CVD risk, level of intellectual disability and sex; which is important in a feasibility study, because they should be representative of the intended population (Thabane et al., 2010). The number of participants in this study was sufficient to answer our research question on whether vigorous RT is feasible for adults with mild to moderate intellectual disabilities and CVD risk factors. Eight participants did report a positive difference in their daily life after the RT‐programme. For example, one participant mentioned that walking was easier, another one said that cycling to work was easier and another participant could better lift the heavy boxes at work.

There are some limitations to this study. The small number of participants and the diversity in age and risk factors limits the generalization of these findings to all adults with intellectual disabilities. It was therefore also not possible to perform subgroup analyses. There might be a selection bias in our sample because these participants were willing to perform the RT‐programme, limiting the generalization of the results to all adults with intellectual disabilities. However, most participants were not already exercising or even familiar with RT‐training. The participant's experiences were derived through a questionnaire that we self‐constructed and were therefore not based on an existing questionnaire. Also because of the self‐report, participants might have provided more favourable answers; therefore, results should be interpreted with caution. Finally, it remains to be determined if the RT‐programme can increase muscle strength and if it can positively influence ADL‐performance and/or CVD risk factors in adults with intellectual disabilities. Future studies should therefore make efforts to increase the total number of training sessions with vigorous training intensity (for example by training longer, use less phases to reach vigorous intensity after familiarization), thereby increasing the potential health benefits for adults with intellectual disabilities and CVD risk factors. Furthermore, future studies should also focus on an RT‐programme for adults with severe and profound intellectual disabilities and on adults with physical limitations who were excluded in this study.

## 5 CONCLUSION

It is feasible for the majority of adults with intellectual disabilities with CVD risk factors to exercise at vigorous intensity. Physiotherapists, physical activity instructors or fitness instructors experienced with working with people with intellectual disabilities can use this RT‐programme to train at vigorous intensity in daily practice for adults with intellectual disabilities, yet close supervision remains necessary during exercising. Vigorous intensity RT seems a promising non‐pharmaceutical new option in the prevention of CVD in adults with intellectual disabilities.

## ETHICAL APPROVAL

The medical ethics committee of the Erasmus Medical Center at Rotterdam, the Netherlands. Approval number: MEC‐2016‐574*.*
